# Assessment and clinical course of hypocalcemia in critical illness

**DOI:** 10.1186/cc12756

**Published:** 2013-06-04

**Authors:** Tom Steele, Ruwanthi Kolamunnage-Dona, Colin Downey, Cheng-Hock Toh, Ingeborg Welters

**Affiliations:** 1 Institute of Ageing and Chronic Disease, University of Liverpool, Daulby Street, Liverpool L69 3GA, UK; 2 Department of Biostatistics, Institute of Translational Medicine, University of Liverpool, Crown Street, Liverpool L69 3BX, UK; 3 Department of Blood Sciences, Royal Liverpool University Hospital, Prescot Street, Liverpool L3 5PS, UK; 4 Institute of Infection and Global Health, University of Liverpool, West Derby Street, Liverpool L69 7BE, UK; 5 Intensive Care Unit, Royal Liverpool University Hospital, Prescot Street, Liverpool L3 8XP, UK

**Keywords:** Electrolyte disorders, Ionized calcium, Adjusted calcium, Hypocalcemia, Critical illness, Intensive care

## Abstract

**Introduction:**

Hypocalcemia is common in critically ill patients. However, its clinical course during the early days of admission and the role of calcium supplementation remain uncertain, and the assessment of calcium status is inconsistent. We aimed to establish the course of hypocalcemia during the early days of critical illness in relation to mortality and to assess the impact of calcium supplementation on calcium normalization and mortality.

**Methods:**

Data were collected on 1,038 admissions to the critical care units of a tertiary care hospital. One gram of calcium gluconate was administered intravenously once daily to patients with adjusted calcium (AdjCa) <2.2 mmol/L. Demographic and outcome data were compared in normocalcemic (ionized calcium, iCa, 1.1-1.3 mmol/L) and mildly and severely hypocalcemic patients (iCa 0.9-1.1 mmol/L and <0.9 mmol/L, respectively). The change in iCa concentrations was monitored during the first four days of admission and comparisons between groups were made using Repeated Measures ANOVA. Comparisons of normalization and outcome were made between hypocalcemic patients who did and did not receive calcium replacement according to the local protocol. The suitability of AdjCa to predict low iCa was determined by analyzing sensitivity, specificity and receiver operating characteristic (ROC) curves. Multivariate logistic regression was performed to determine associations of other electrolyte derangements with hypocalcemia.

**Results:**

55.2% of patients were hypocalcemic on admission; 6.2% severely so. Severely hypocalcemic patients required critical care for longer (*P *= 0.001) compared to normocalcemic or mildly hypocalcemic patients, but there was no difference in mortality between groups (*P *= 0.48). iCa levels normalized within four days in most, with no difference in normalization between those who died and survived (*P *= 0.35). Severely hypocalcemic patients who failed to normalize their iCa by day 4 had double the mortality (38% *vs*. 19%, *P *= 0.15). Neither iCa normalization nor survival were superior in hypocalcemic patients receiving supplementation on admission. AdjCa <2.2 mmol/L had a sensitivity of 78.2% and specificity of 63.3% for predicting iCa <1.1 mmol/L. Low magnesium, sodium and albumin were independently associated with hypocalcemia on admission.

**Conclusions:**

Hypocalcemia usually normalizes within the first four days after admission to ICU and failure to normalize in severely hypocalcemic patients may be associated with increased mortality. Calcium replacement appears not to improve normalization or mortality. AdjCa is not a good surrogate of iCa in an ICU setting.

## Introduction

Hypocalcemia is a common derangement in both medical and surgical patients requiring intensive care. The reported prevalence varies significantly between studies due to differences in the population studied and the cutoff values used, with published figures ranging from 15% to 88% [1,2].

The etiology of hypocalcemia in critical illness is multifactorial [3,4]. Abnormalities of parathyroid hormone secretion and action as well as vitamin D deficiency, medication side effects, transfusion of citrated blood and the effect of circulating catecholamines are likely to contribute [1,4-7]. Septic patients are at particular risk of hypocalcemia and this has been linked to both the bacteremic state and the effect of inflammatory mediators on parathyroid hormone secretion and function [4,8,9].

A number of studies have reported that hypocalcemia is associated with poor outcome in different populations including medical, surgical and trauma patients on both adult and pediatric intensive care units [2,10-15]. However, results from recent large studies suggest that it is only severe hypocalcemia (ionized calcium <0.8 mmol/L or 0.9 mmol/L) that is independently associated with mortality [16-18].

It has been advocated that calcium should be replaced in order to prevent life-threatening complications such as laryngospasm, tetany, seizures and cardiac abnormalities [19]. However, studies using experimental models of sepsis have shown calcium supplementation to provide either no benefit or even to increase mortality [20,21].

There is no evidence on the impact of calcium supplementation on outcome in critically ill patients. A recent systematic review failed to identify any studies that evaluated the associations of parenteral calcium supplementation in intensive care patients and outcomes such as length of stay and mortality [20]. In addition, neither the clinical course of hypocalcemia nor the relationship of normalization to outcome has been established. Hence it remains controversial whether or not calcium replacement should be routinely given to critically ill patients and a more complete understanding of both the course of hypocalcemia in relation to outcome and the effect of supplementation is needed.

The assessment of calcium status on the intensive care unit (ICU) is also inconsistent. Approximately 50% of total circulating calcium is ionized, which is the biologically active form. Of the remainder, around 40% is bound to plasma proteins, particularly albumin, and a further 10% to anions such as phosphate, sulphate and citrate [22]. As measurements of total calcium are traditionally more accessible than those of ionized calcium, formulas are used to correct the total calcium concentration for variations in albumin concentration in order to reflect ionized calcium concentrations. However, several factors can alter the proportion of total calcium that is ionized and studies have shown that adjusted calcium formulae do not provide a reliable substitute for ionized calcium in various groups of critically ill patients [23-25]. Despite this, adjusted calcium continues to be used in many ICUs to guide clinical practice.

Other biochemical abnormalities are also common in critically ill patients, with derangements of sodium, potassium, magnesium, phosphate, lactate and albumin frequently seen [26]. Magnesium and phosphate concentrations are known to affect calcium homeostasis, however there has been little study of the interaction of these abnormalities in the context of hypocalcemia and greater understanding could improve the therapeutic approach to hypocalcemic patients[3].

We retrospectively reviewed data from 1,038 admissions to an ICU in which calcium supplementation is routinely given, in this case guided by the patients' adjusted calcium level. The primary objectives were to establish the clinical course of hypocalcemia early in admission and the relationship of this to outcome, and to assess the impact of calcium supplementation on normalization of calcium concentration and outcome. In addition, we determined the suitability of using adjusted calcium levels in place of ionized calcium to guide clinical practice and examined the association of various biochemical derangements with hypocalcemia.

## Materials and methods

This was a single-center, retrospective, observational study. The population is derived from patients prospectively recruited to a non-interventional study assessing a biomarker of infection in a full cross-section of intensive care patients. Patients admitted to the ICU, the postoperative critical care unit or the high dependency unit at Royal Liverpool University Hospital between January 2008 and January 2012 were eligible for inclusion. Ethical approval was obtained from the Bolton Research Ethics Committee (study 07/H1009/64) and consent was obtained from all participants. Patients were excluded if valid consent could not be obtained, if they were discharged, died or put on the care of the dying pathway within the first 48 h or if they were admitted after elective surgery.

Admission details, demographic information and the Acute Physiology and Chronic Health Evaluation (APACHE) II score were collected and patients followed up to determine 28-day mortality. A senior clinician assessed the patient records to determine sepsis status on a daily basis retrospectively according to the 1992 ACCP/SCCM consensus guidelines [27]. All blood tests were carried out as part of routine clinical practice and consisted of daily serum chemistry and 4-hourly blood gas analysis unless otherwise dictated by clinical need. Results were retrieved electronically from the hospital laboratory records and information was held on a study database. Admission serum biochemistry and blood gas results (defined as the first available results within 24 h of the recorded admission date) were extracted from the database alongside the serum biochemistry and blood gas results at 6 am (+/- 2 hours) for the 3 days following admission. Missing results were traced by searching patient records electronically, or manually if necessary.

Ionized calcium (iCa), pH and lactate results were obtained from blood gas analysis on whole arterial blood collected in heparinized syringes. Measurements were carried out by trained staff on a RapidLab 1265 blood gas analyzer (Siemens Healthcare Diagnostics, Inc.) based on the ICU. iCa was not adjusted for pH. Sodium and potassium were measured using ion-selective electrodes using the Roche/Hitachi cobas 8000 ISE analyzer module in the central hospital laboratory. Total calcium, magnesium, phosphate and albumin measurements were carried out with dye-forming reactions detected photometrically using the Roche/Hitachi cobas c701 analyzer module in the central hospital laboratory. Adjusted calcium (AdjCa) is calculated in this hospital by a locally defined equation based on the relationship between albumin and total calcium concentrations [28]: AdjCa (mmol/L) = Total Calcium (mmol/L) + 0.133(44.1-Albumin (g/L)). Other published formulas for the adjustment of calcium were also used to calculate AdjCa values for each patient. The reference range for AdjCa was 2.2 to 2.6 mmol/L and for iCa it was 1.1 to 1.3 mmol/L.

**Figure 1:**
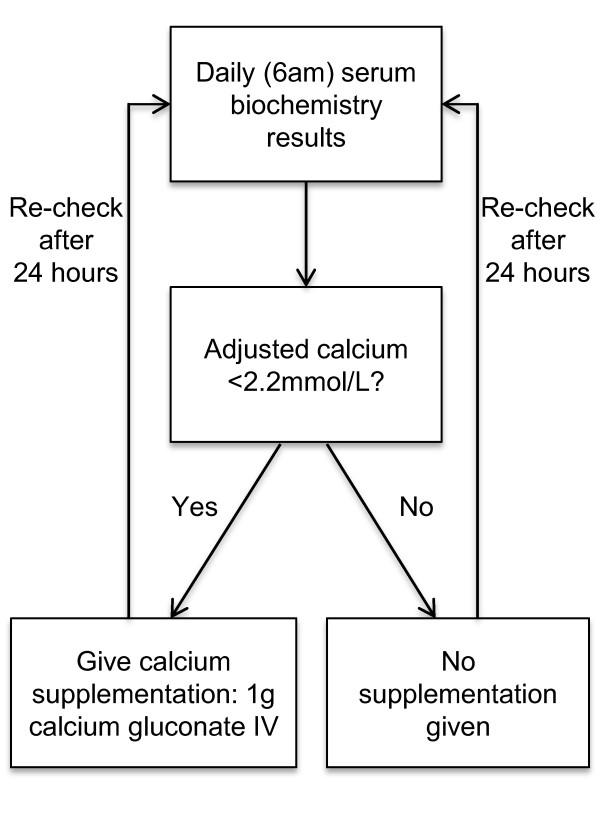
**Flow chart outlining calcium supplementation protocol followed on units in which the study was carried out**.

The protocol for calcium supplementation is outlined in Figure 1. One gram of calcium gluconate was administered to all patients with AdjCa <2.2 mmol/L as 10 mL 10% Calcium Gluconate, diluted in 20 mL of normal saline, given intravenously over 30 min. This was carried out once daily based on the morning biochemistry analysis for as long as the AdjCa remained <2.2 mmol/L.

All statistical analysis was performed using SPSS version 20 for Windows (IBM). Patients hypercalcemic on admission were excluded from analyses. Continuous data were assessed for normality visually by plotting histograms. For normally distributed data the mean (standard deviation) and parametric tests were used and for non-normally distributed data the median (interquartile range (IQR)) and non-parametric tests were used. Categorical data, quoted as *n*(%), were assessed using cross-tabulation and the chi squared test for independence. A *P *value < 0.05 was taken to be statistically significant. Bonferroni correction was performed where multiple analyses were carried out within dependent hypotheses.

The mean 6 am iCa concentration on each day post admission was calculated to create a time profile of iCa over the first 4 days of admission. Repeated measures ANOVA was carried out to assess the effect of time post admission on iCa concentration and the influence of between group factors on the time effect. The Greenhouse-Geisser adjustment was used to generate F and *P *values.

The diagnostic sensitivity, specificity, positive predictive value (PPV) and negative predictive value (NPV) value of using AdjCa <2.2 mmol/L (derived from the laboratory and from other published equations) in predicting iCa <1.1 mmol/L was assessed. The discriminative power of AdjCa for predicting iCa was evaluated by producing receiver operator characteristic (ROC) curves and by calculating the area under the curve (AUC).

The association between other biochemical derangements and hypocalcemia was assessed by multivariate logistic regression using iCa <1.1 mmol/L as a binary outcome. Relevant biochemical factors (sodium, potassium, phosphate, magnesium, lactate and albumin) and potential confounding demographic predictors (age, sex, APACHE II score, sepsis status) with *P *< 0.25 from the univariate analysis were included in multivariate logistic regression model to assess the association with hypocalcemia. Biochemical factors, age and APACHE II score were entered into the model as continuous linear predictors and sex and sepsis status as categorical predictors.

## Results

Data from 1,038 admissions were available for inclusion in the study. Demographic information is displayed in Table 1. The median age on admission was 61 years (IQR 48-73), 56.8% of patients were men and the median APACHE II score was 18 (IQR 13-23). An iCa measurement within 24 h of admission was available for 976 (94%) patients. The mean admission iCa was 1.08 mmol/L (SD 0.11) and 539 (55.2%) patients were classified as hypocalcemic (iCa <1.1 mmol/L). Further subclassification revealed that 60 (6.1%) patients were severely hypocalcemic on admission (iCa <0.9 mmol/L), 479 (49.1%) were mildly hypocalcemic (0.9-1.09 mmol/L) and 420 (43%) were normocalcemic (1.1-1.3 mmol/L). Seventeen (1.7%) were hypercalcemic (>1.3 mmol/L) and excluded from further analysis. There was no significant difference in mortality between severely hypocalcemic, mildly hypocalcemic and normocalcemic patients (23.3% *vs*. 19.4% *vs*. 17.4%, respectively (*P *= 0.477, chi squared test)). There was, however, a significant difference in length of stay (14 *vs*. 9 *vs*. 8 days; *P *= 0.001, Kruskal Wallis test).

**Table 1:** Baseline variables for all patients and for those normocalcemic and hypocalcemic.

Variable	All patients	Normocalcaemic (*n *= 420)	Hypocalcaemic (*n *= 539)	*P *value
Age (years)	61 (48-73)	63 (47-73)	61 (49.5-72)	0.83
Male sex	590 (56.8)	246 (58.6)	298 (55.3)	0.31
APACHE II score	18 (13-23)	18 (13-23)	19 (14-23)	0.18
Septic during first 3 days	531 (51.2)	199 (47.4)	308 (57.2)	0.002
				
Alive at 28 days	848 (81.7)	347 (82.6)	432 (80.1)	0.33
Length of stay (days)	8 (4-16)	8 (4-15.5)	9 (4-19)	0.04
				
pH	7.38 (7.3-7.44)	7.38 (7.31-7.44)	7.37 (7.3-7.44)	0.54
Sodium	138 (134-141)	138 (135-144)	137 (133-141)	0.015
Potassium	4.2 (3.8-4.6)	4.2 (3.8-4.6)	4.2 (3.8-4.7)	0.6
Phosphate	1.19 (0.91-1.53)	1.14 (0.91-1.44)	1.27 (0.91-1.61)	0.003
Magnesium	0.78 (0.66-0.89)	0.8 (0.69-0.89)	0.75 (0.64-0.89)	0.002
Lactate	1.5 (1-2.5)	1.4 (1-2.3)	1.7 (1.1-2.8)	<0.001
Albumin	24 (18-30)	26 (20-33)	21 (21-26)	<0.001

Values represent total (%) for categorical variables and median (interquartile range) for continuous variables. Mann-Whitney U test used for continuous variables, chi-squared test for categorical.

APACHE2, Acute Physiology and Chronic Health Evaluation II score.

### Change in ionized calcium level over the first 4 days of admission

**Figure 2:**
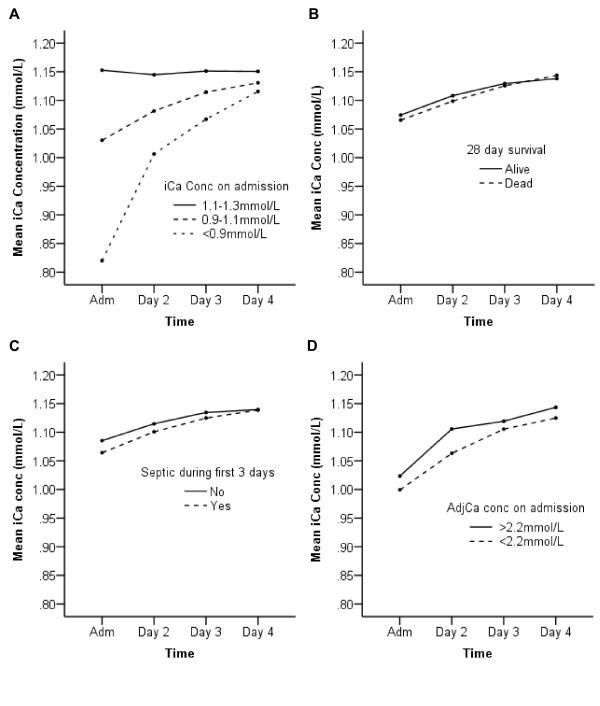
**Time course of mean ionized calcium concentrations over the first 4 days of admission**. The mean ionized calcium concentrations for various population subgroups at admission and at 06:00 for the following 3 days are plotted. Repeated measures ANOVA was carried out for linear changes in iCa over the period. F and *P *values are based on the Greenhouse-Geisser adjustment. **(A) **Changes in mean ionized calcium concentrations for normocalcemic, mildly hypocalcemic and severely hypoclacemic patients. Time effect F = 273.6; *P *< 0.001, effect of calcium status on time effect F = 102.7; *P *< 0.001. **(B) **Comparison of changes in mean iCa concentration for patients that survived and those that died. Time effect F = 82.4; *P *< 0.001, effect of survival group on time effect F = 1.06; *P *= 0.35. **(C) **Comparison of changes in iCa between those who did and did not have sepsis within 3 days of admission as determined by a senior consultant using APPC/SCCM consensus guidelines. Time effect F = 99.2; *P *< 0.001, effect of sepsis status on time effect F = 2.22; *P *= 0.058. **(D) **Comparison of changes in iCa levels in patients with ionised hypocalcemia on admission who also had adjusted calcium <2.2 mmol/L and therefore received calcium supplementation and those who did not. Time effect F = 135.1; *P *< 0.001, effect of adjCa status on time effect F = 1.86; *P *= 0.15.

To study how ionized calcium concentration changed over the first 4 days of admission to ICU, the mean of iCa measurements taken on admission and at 06:00 on days 2, 3, and four were plotted (Figure 2). By the fourth day of their stay the mean level for patients with severe and mild hypocalcemia on admission had normalized (Figure 2A). Of those severely hypocalcemic on admission, 24 (53%) had an iCa <1.1 mmol/L on day 4, compared to 121 (35.2%) of those mildly hypocalcemic and 64 (21.9%) of those normocalcemic (*P *= 0.001, Table 2). There was no difference in the influence of time on iCa concentration over the first 4 days between those who survived and those who died. Also, no difference was found between patients with and without sepsis, as confirmed by repeated measures ANOVA (*P *= 0.35 and *P *= 0.1 respectively; Figure 2B and 2C).

**Table 2:** Prevalence of hypocalcaemia on day 4 and mortality in different patient subgroups.

Subgroup	Day 4 iCa (mmol/L)	*n *(%)	*P *value^a^	Mortality	*P *value^b^
All patients	<1.1	210 (30)		46 (21.9)	
	>1.1	489 (70)	-	102 (20.9)	0.76
					
Admission iCa <0.9 mmol/L	<1.1	24 (53.3)		9 (37.5)	
	>1.1	21 (46.7)		4 (19)	0.15*
Admisson iCa 0.9-1.09 mmol/L	<1.1	121 (35.2)		25 (20.7)	
	>1.1	223 (64.8)		50 (22.4)	0.71*
Admission iCa 1.1-1.3 mmol/L	<1.1	64 (21.9)		12 (18.8)	
	>1.1	228 (78.1)	0.001	45 (19.7)	0.86*
					
Hypocalcemia & sepsis	<1.1	98 (38.1)		24 (24.5)	
	>1.1	159 (61.9)		34 (21.4)	0.56**
Hypocalcemia & no sepsis	<1.1	47 (35.6)		10 (21.3)	
	>1.1	85 (64.4)	0.63	20 (23.5)	0.77**
					
Admission iCa <1.1 mmol/L: Survivors	<1.1	111 (36.9)		0	
	>1.1	190 (63.1)		0	
Admission iCa <1.1 mmol/L: Non-survivors	<1.1	34 (38.6)		100	
	>1.1	54 (61.4)	0.76	100	-
Adm iCa <1.1 & AdjCa <2.2 mmol/L (given supplementation)	<1.1	121 (39.7)		29 (24.0)	
	>1.1	184 (60.3)		42 (22.8)	0.82**
Adm iCa <1.1 & AdjCa >2.2 mmol/L (not given supplementation)	<1.1	24 (28.9)		5 (20.8)	
	>1.1	59 (71.1)	0.073	12 (20.3)	0.96**

Mortality is reported as n (%). All *P *values are derived from the chi-squared test for independence between groups.

^a^Difference in % patients with iCa >1.1 mmol/L by day 4 between subgroups.

^b^Difference in mortality by day 4 iCa status within each subgroup.

Bonferroni correction was performed where multiple subgroup analyses were carried out; *P < 0.017, **P < 0.025 required for statistically significant result.

AdjCa, Adjusted calcium; iCa, Ionized calcium.

Subsequently, the mortality rates of patients who did and did not normalize their iCa level by day 4 were compared by cross-tabulating results and using the chi-squared test for independence (Table 2). Overall, 30% of patients were hypocalcemic on day 4 and there was no difference in mortality between these patients and those who were not (*P *= 0.76). Over half of patients with severe hypocalcemia on admission failed to normalize iCa over the first 4 days, whereas 65% of mildly hypocalcemic patients had normal iCa on day 4. There was an increased mortality in those patients who were severely hypocalcemic on admission and had iCa <1.1 mmol/L on day 4 (37.5% *vs*. 19%), although this was not statistically significant (*P *= 0.15), possibly due to the small sample size (*n *= 24 with day 4 iCa <1.1 mmol/L and *n *= 21 with day 4 iCa >1.1 mmol/L). In patients with mild hypocalcemia on admission there was no difference in mortality between those who did and did not normalize their iCa level (*n *= 223 and *n *= 121 respectively; *P *= 0.71). Furthermore, the proportion of patients with normal iCa on day 4 was similar in survivors and non-survivors (63.1 *vs*. 61.4%; *P *= 0.76) and in those with and without sepsis (61.9% *vs*. 64.4%; *P *= 0.63).

### Impact of calcium supplementation on normalization of hypocalcemia and survival

Adjusted calcium (AdjCa) was available on admission for 1,031 (99.3%) patients and the median level was 2.17 mmol/L (IQR 2.07-2.25). 602 (58%) were classified as hypocalcemic (<2.2 mmol/L) and met criteria for calcium supplementation, 416 (40.3%) were normocalcemic and 13 (1.3%) were hypercalcemic (>2.6 mmol/L). Of patients with hypocalcemia as defined by the gold standard (iCa <1.1 mmol/L), 419 (78%) had AdjCa <2.2 mmol/L, and thus received calcium supplementation in accordance with the local protocol. The overall survival in this group was no different to those with AdjCa <2.2 mmol/L not meeting criteria for supplementation (80.2% and 81.6%, respectively, *P *= 0.12). In addition, there was no difference in the median increase in iCa levels over the first 24 h between hypocalcemic patients receiving supplementation and those who did not (0.06 mmol/L (0.02-0.12) and 0.06 mmol/L (0.01-0.11), respectively, *P *= 0.35). The results were similar when looking across the full 4 days (0.11 mmol/L (0.04-0.18) and 0.1 mmol/L (0.03-0.16), respectively; *P *= 0.34). Repeated measures ANOVA showed that the effect of time on iCa level was not significantly different between those who did and did not receive supplementation on admission (*P *= 0.15, Figure 2D).

### Adjusted calcium for guiding therapeutic practice on the ICU

**Figure 3:**
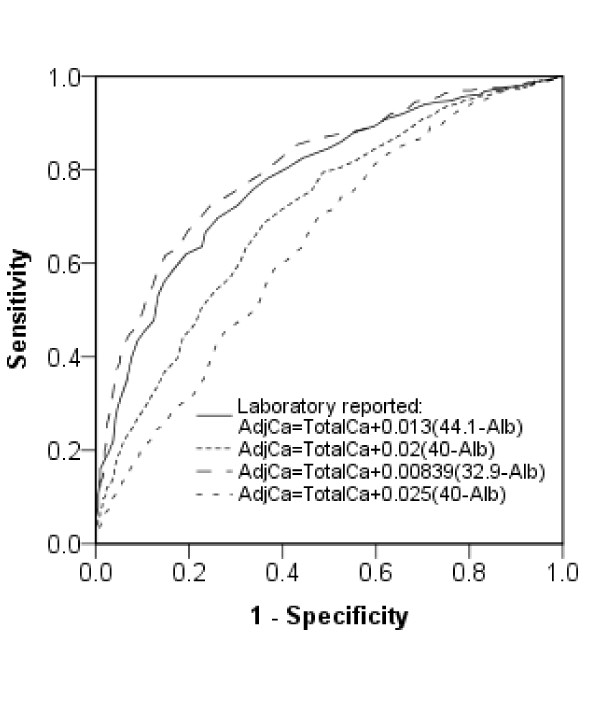
**ROC curves for albumin-adjusted calcium in predicting iCa <1.1**. Receiver operating characteristic curves displaying the performance of albumin-adjusted calcium in predicting hypocalcemia. Different lines represent different formulae used, locally defined and in the literature [23]. Hypocalcemia is defined as iCa <1.1 mmol/L.

The local AdjCa cutoff of 2.2 mmol/L had a sensitivity of 78.2% and specificity of 63.3% for detecting iCa <1.1 mmol/L in this population (Table 3). Other formulas for adjusting total calcium levels were also assessed but none could give both a high sensitivity and specificity at the cutoff values used (Table 3). The largest area under the curve (0.81; 95% CI 0.78-0.83) came from the formula AdjCa=Total Ca+0.00839(32.9-Alb) (Figure 3). Due to the potential importance of severe hypocalcemia, the ability of AdjCa to predict this was also assessed. AdjCa <2 mmol/L, derived from the locally validated formula, had a sensitivity of 60%, a specificity of 89% and a PPV of just 27% for detecting iCa <0.9 mmol/L. The sensitivity and specificity of AdjCa <2.2 mmol/L for predicting iCa <1.1 mmol/L did not improve in patients with normal pH (74.9% and 67.7%, respectively) or normal serum phosphate (80.3% and 58.8%, respectively).

**Table 3:** The performance of locally validated and published formulas for adjusted calcium in predicting hypocalcemia.

Formula		Sens (%)	Spec (%)	PPV	NPV	AUC (95% CI)
Locally Validated	AdjCa=Total Ca+0.013(44.1-Alb)	78.2	63.3	72.4	70.2	0.78 (0.75-0.81)
Traditionally quoted [24]	AdjCa=Total Ca+0.02(40-Alb)	60.4	69.0	70.6	58.7	0.71 (0.68-0.74)
Slomp et al. [24]	AdjCa=Total Ca+0.025(40-Alb)	32.3	78.9	65.4	48.6	0.64 (0.61-0.68)
Slomp et al. [24]	AdjCa=Total Ca+0.00839(32.9-Alb)	97.4	16.7	59	83.9	0.81 (0.78-0.83)

Hypocalcemia defined as iCa <1.1 mmol/L, and AdjCa cutoff was 2.2 mmol/L.

AdjCa, adjusted calcium (mmol/L); Alb, Albumin concentration (g/L); AUC, area under the curve; NPV, negative predictive value; PPV, positive predictive value; Sens, sensitivity; Spec, specificity; Total Ca, Total calcium (mmol/L).

### Association of biochemical derangements and hypocalcemia

Univariate analysis indicated that the proportion of patients who developed sepsis within 3 days of admission differed significantly between hypocalcemic and normocalcemic patients (57.2% *vs*. 47.4%; *P *= 0.002; Table 1). There were also significant differences between the median admission sodium, phosphate, magnesium, lactate and albumin between the groups (Table 1). On multivariate logistic regression analysis lower admission sodium (odds ratio 0.96 (95% CI 0.94-0.99)), magnesium (0.37 (95% CI 0.17-0.81)), and albumin (0.93 (95% CI 0.91-0.94)) were significantly associated with low iCa, as was raised lactate (1.11 (95% CI1.03-1.2). Low admission phosphate showed a trend, but was not significantly associated with low iCa (*P *= 0.052; Table 4).

**Table 4:** Multivariate logistic regression analysis for biochemical abnormalities with hypocalcemia.

Explanatory variable	OR (95% CI)	*P *value
APACHE II	1.00 (0.98-1.02)	0.72
Sepsis (first 3 days)	1.03 (0.76-1.38)	0.87
Sodium	0.96 (0.94-0.99)	0.002
Phosphate	1.30 (1.00-1.69)	0.052
Magnesium	0.37 (0.17-0.81)	0.013
Lactate	1.11 (1.03-1.20)	0.007
Albumin	0.93 (0.91-0.94)	<0.001

Variables entered into model were those with *P *< 0.25 on univariate analysis. APACHE II, sodium, phosphate, magnesium, lactate and albumin were continuous predictors, entered linearly and sepsis a binary predictor. Outcome was iCa <1.1 mmol/L.

APACHE II, Acute Physiology and Chronic Health Evaluation II score; CI, Confidence interval; OR, Odds ratio (for ionized calcium <1.1 mmol/L).

## Discussion

In this large retrospective, observational study hypocalcemia was a very common abnormality in patients upon admission to intensive care. The prevalence of 55% was slightly lower than recently reported values, which have ranged to beyond 80%, probably reflecting higher cut off values for hypocalcemia [2,16,17]. We chose an ionized calcium concentration of 1.1 mmol/L as cutoff, as this is the lower reference value in our hospital. Other papers have used values lower than this, highlighting the difficulty in comparing prevalence figures [15].

In contrast to several previous reports we found no significant association between hypocalcemia on admission and mortality [10-15]. Several authors have described a significantly higher mortality rate in severely hypocalcemic patients in particular and suggested that it is in this group in which the association is clinically significant [16-18]. We could not confirm this, despite a trend in that direction, probably due to the small number of severely hypocalcemic patients in our study. However, in our study, patients with low ionized calcium had a longer stay on the ICU, and this finding was most prominent in severely hypocalcemic patients. It is uncertain whether this is a causal relationship as severely hypocalcemic patients may represent a more severely ill subset of intensive care patients, requiring longer stays. Alternatively, the diagnoses of severely hypocalcemic patients may differ from other groups, although assessing this possibility is beyond the scope of this study.

To our knowledge, this is the first study to describe the time course of calcium levels over the early days of admission to intensive care. We found that both mild and severe hypocalcemia shows sustained improvement over the first 4 days of admission. There was no difference in the pattern of change of ionized calcium concentrations in survivors and non-survivors. Furthermore, in patients with mild hypocalcemia on admission, there was no difference in mortality between those who did and did not normalize their ionized calcium by day 4 post admission. However, among patients with severe hypocalcemia on admission, there was a notable but not statistically significant increase in mortality in those who had failed to normalize their ionized calcium levels by day 4. The failure to reach statistical significance was most likely due to the small number of patients who were severely hypocalcemic on admission and in whom calcium levels were available on day 4. It appears that in patients with mild hypocalcemia on admission, normalization of ionized calcium level does not have an influence on mortality. However, in patients with severe hypocalcemia, normalization of calcium concentrations may be less likely to occur and mortality may be higher in this group, suggesting they could be targets for intervention.

Our study is set apart from others in that many participants received intravenous calcium supplementation as part of a standard protocol. This was guided by adjusted rather than ionized calcium, allowing analysis of whether this practice affected the normalization of ionized calcium concentration and outcome. We found that the pattern of change over time and the proportion of hypocalcemic patients with a normal ionized calcium concentration by day 4 were not different between those who did and did not receive calcium supplementation. Our results suggest that calcium supplementation does not lead to a greater sustained increase in ionized calcium than occurs without supplementation. This is in keeping with previous reports suggesting that calcium supplementation does not increase ionized calcium concentrations in critical illness and that calcium concentrations cannot be corrected by calcium therapy alone [20,29,30]. However, it needs to be taken into consideration that our study was retrospective and observational and hence lacks controlled intervention.

Normally, ionized calcium concentrations are tightly regulated and hypocalcemia can have profound adverse clinical effects [19]. Furthermore, intravenous calcium has an important role in hemodynamic support [31]. Interestingly, however, it has been shown that in sepsis and during treatment with catecholamines calcium is shifted into the cells, which may contribute to the hypocalcemia seen in the critically ill [7,32,33]. Raised intracellular calcium concentrations have in turn been linked to the pathophysiology of sepsis [33]. Giving additional parenteral calcium could therefore cause harm, a concept which is supported by experimental models of sepsis in which calcium administration was associated with higher mortality [20,21]. In keeping with this, it has frequently been suggested that hypocalcemia is a marker of disease severity, rather than a contributor in itself, indicating therapy is unwarranted [17,22].

Despite this, the role of calcium replacement in hypocalcemic patients remains unclear and requires further study. The results of this investigation are limited as the study design was retrospective and purely observational, no controlled intervention was performed and not all confounders could be eliminated. In particular, some patients may have received calcium supplementation prior to admission to intensive care and the route of nutrition (enteral *vs*. parenteral) was not included in the analysis. Finally, although the overall sample size was large, the rarity of severe hypocalcemia renders the study underpowered to make conclusions in this subgroup.

As a secondary finding, this study confirmed that a low adjusted calcium concentration is a relatively poor surrogate for identifying critically ill patients with low ionized calcium concentrations, despite its widespread use for this purpose. The adjustment of total calcium is based on a formula incorporating the relationship between total calcium and albumin, derived from the linear regression equation and the difference of the patient's albumin from the population average [28]. In the present study, a formula developed from results of patients in our hospital has been used and therefore neither epidemiological factors nor differences in equipment could account for the limitations described.

There are a number of plausible physiological explanations for adjusted calcium being a poor predictor for ionized calcium in critically ill patients. They include alterations in pH affecting calcium-albumin binding, deranged serum concentrations of phosphate, which chelates calcium, or changes in blood concentrations of citrate and fatty acids [23,34]. We found that low adjusted calcium remained a poor predictor of low ionized calcium, even when looking solely at patients without derangements of pH and phosphate concentration. As a result we propose that, wherever available, measurement of ionized calcium rather than adjusted calcium should be performed to define hypocalcemia. Only if ionized claclium cannot be measured, adjusted calcium should be calculated, with the formula published by Stomp providing the best area under the curve [23].

The finding that lower magnesium, sodium and albumin were independently associated with ionized hypocalcemia, with a strong trend for higher phosphate, suggests these biochemical derangements should be specifically considered in the assessment of hypocalcemic patients. This is unsurprising as magnesium deficiency in particular is a known cause of hypocalcemia due to impaired parathyroid hormone secretion and end organ resistance [3,35].

## Conclusions

Hypocalcemia is a common finding in patients admitted to a general ICU. In this large cohort of patients, levels tended to normalize over the early days of admission and this was not associated with outcome. However, patients severely hypocalcemic on admission whose calcium failed to rebound to normal trended towards having a higher mortality and may be a target for intervention. Findings also indicate that calcium supplementation is not associated with calcium normalization or improved outcome, although this study was observational in nature. A large randomized controlled trial of calcium supplementation in critically ill patients with different degrees of hypocalcemia is required to determine the effectiveness and side effects of this treatment and its role in clinical practice.

## Key messages

• Hypocalcaemia is common in critical illness but tends to normalize during the early days of admission to intensive care.

• In most cases normalization of calcium status is not associated with improved outcome, however mortality may be higher in severely hypocalcaemic patients who remain hypocalcemic on the fourth day of their admission.

• Calcium supplementation does not improve either calcium normalization or survival and a large randomized controlled trial is required to assess the effectiveness and side effects of this intervention.

• Albumin-adjusted calcium is a poor predictor of low ionized calcium in critical illness and should not be used in this setting.

• Low magnesium, sodium and albumin are independently associated with low ionized calcium and this should be considered in the overall management of each patient.

## Abbreviations

AdjCa: Adjusted calcium; ANOVA: Analysis of variance; APACHE: Acute Physiology and Chronic Health Evaluation; AUC: Area under the curve; CI: Confidence interval; iCa: Ionized calcium; ICU: Intensive care unit; IQR: Interquartile range; NPV: Negative predictive value; OR: Odds ratio; PPV: Positive predictive value; ROC: Receiver operating characteristic; SD: Standard deviation.

## Competing interests

The authors declare that they have no competing interests.

## Authors' contributions

TS carried out the statistical analysis and drafted the manuscript. RKD helped design the study and directed and assisted the statistical analysis. CD collected data and designed and managed the database. CHT conceived and designed the initial study, amended the manuscript and was responsible for conducting the study. IW conceived and designed the study, coordinated the study, oversaw data collection and revised the manuscript. All authors helped revise the manuscript critically and have read and approved the final manuscript for publication.
